# Advancing quantitative outcome measurement in therapeutic dance

**DOI:** 10.3389/fpsyg.2026.1749839

**Published:** 2026-05-12

**Authors:** Judith Bek, Deborah A. Jehu, Gammon M. Earhart, Madeleine E. Hackney

**Affiliations:** 1 School of Psychology, University College Dublin, Dublin, Ireland; 2 Centre for Motor Control, Faculty of Kinesiology and Physical Education, University of Toronto, Toronto, ON, Canada; 3 Department of Community and Behavioral Health Sciences, Institute of Public and Preventive Health, School of Public Health, Augusta University, Augusta, GA, United States; 4 Program in Physical Therapy, Department of Neurology, Washington University in St. Louis School of Medicine, St. Louis, MO, United States; 5 Department of Neuroscience, Washington University in St. Louis School of Medicine, St. Louis, MO, United States; 6 Department of Medicine, Division of Geriatrics and Gerontology, Emory University School of Medicine, Atlanta, GA, United States; 7 Atlanta VA Center for Visual and Neurocognitive Rehabilitation, Atlanta, GA, United States; 8 Birmingham/Atlanta VA Geriatric Research Education and Clinical Center, Atlanta, GA, United States

**Keywords:** artificial intelligence—AI, biomechanics, dance therapy, digital health, motion capture (mocap), neuroimaging, neurorehabilitation, telemedicine

## Dance as a therapeutic approach

Dance can offer multiple physical and mental health benefits, and community dance programs aiming to promote health and wellbeing are widely available. Such therapeutic dance programs have been studied across populations, including individuals with dementia, Parkinson's disease (PD), stroke, cancer, autism spectrum disorder, and neurotypical older adults (Kshtriya et al., 2015; Patterson et al., 2018; Takahashi et al., 2019; Bek et al., 2020; Ares-Benitez et al., 2022; Wang et al., 2022; Karkou et al., 2023). Therapeutic dance classes can be offered online, enabling larger numbers of people to potentially benefit, particularly those with limited access to in-person groups or travel-related barriers (Bek et al., 2021, 2022, 2025; Ghanai et al., 2021).

Health outcomes of therapeutic dance are typically assessed using a wide variety of general and population-specific measures. Limitations of current measurement tools may contribute to inconsistent findings in the literature, masking the true potential of dance to induce health benefits (Hwang and Braun, 2015; Shanahan, 2015; Bek et al., 2020; Carapellotti et al., 2020). Advances in digital technology and artificial intelligence (AI) offer significant potential to obtain detailed, quantitative measures of movement in both in-person and remote contexts, providing new opportunities to precisely evaluate therapeutic dance outcomes.

This opinion article discusses how emerging technologies that can quantify motor and neuroplastic changes might go beyond conventional outcome measures for therapeutic dance, considers potential challenges of these new approaches, and outlines future directions for research and practice.

## Current measurement approaches for therapeutic dance

Validated clinical measures are often used to assess physical outcomes of dance in clinical populations, including gait (e.g., 6-min or 10-m walk test), balance (Berg balance scale, balance evaluation systems test), and functional mobility (Timed Up and Go, sit-to-stand) (Earhart, 2009; Mattle et al., 2020). Measurement tools designed for specific health conditions are also often used, such as the Unified Parkinson's Disease Rating Scale (UPDRS/MDS-UPDRS) for PD (Goetz et al., 2008) and the Fugl-Meyer Assessment (Fugl-Meyer et al., 1975) for stroke. Self-report questionnaires may also be used to assess physical outcomes such as activities of daily living, dexterity, and falls (e.g., Lee et al., 2015; Blanco-Rambo et al., 2022).

These established measures can be valuable in assessing dance outcomes, and their wide use across interventions can facilitate comparisons between studies and meta-analyses. However, while clinical rating scales and other conventional assessment tools have yielded much useful data on dance outcomes, they are not designed to capture the specific effects of dance. Some further limitations of these tools are considered below.

Subjective rating scales are limited in reliability and sensitivity (e.g., Chan et al., 2018; Hsiao et al., 2021), and some (e.g., UPDRS) require trained professionals to administer. Patient-reported outcomes are valuable to understand participants' experiences from their own perspective, but may be impacted by cognitive impairment or reduced insight, particularly in neurological conditions (e.g., Cameron et al., 2024).

Existing technology-based measures of dance outcomes include instrumented gait mats, treadmills, balance boards, and force plates (e.g., Guzmán-García et al., 2011; Sohn et al., 2018) as well as automated versions of established clinical tests, such as Timed Up and Go and sit-to-stand (Tan et al., 2019; Vourganas et al., 2019). While gait, functional mobility, and balance are important outcomes in terms of falls prevention and mobility, it is also useful to assess upper-body kinematics, including limb movements and fine motor coordination, which may be improved through dance (particularly in seated dance programs designed for people with limited mobility). Potential upper-body outcomes of dance that are not well addressed by current measures include bimanual coordination, sequencing, amplitude, smoothness, and range of motion.

In summary, the beneficial effects of dance may be underestimated because many current measurement techniques are not sufficiently sensitive to change or do not fully capture relevant aspects of movement addressed by dance.

These limitations are further compounded by small sample sizes (e.g., Patterson et al., 2018; Bek et al., 2020; Ares-Benitez et al., 2022). Additionally, few studies have tracked outcomes of therapeutic dance over an extended period to assess ongoing improvement or maintenance of benefits (for reviews, see Sharp and Hewitt, 2014; Kalyani et al., 2019; Bek et al., 2020).

## Emerging solutions for measuring dance outcomes

Technological developments, particularly in remote motion capture and mobile neuroimaging, expand the possibilities for precise and reliable measurement of therapeutic benefits, potentially overcoming some of the limitations discussed above. As technology advances, the ability to measure outcomes not only in controlled clinical or laboratory environments but also in real-world daily living contexts is rapidly expanding.

## Motion capture

Motion capture and wearable motion sensors (inertial measurement units; IMUs) have long been used to assess outcomes of interventions, although few studies used these to test therapeutic effects of dance (Hulbert et al., 2020; Haputhanthirige et al., 2023). These technologies enable more comprehensive measurement of full-body movement than conventional laboratory-based instruments such as gait mats and balance boards.

Traditional motion capture systems, which utilize reflective or infrared markers, are expensive and require time and expertise for setup and analysis. Portable motion capture using depth sensing systems like Kinect (e.g., Sampaio et al., 2016; Garcia-Agundez et al., 2019; Lu et al., 2024), as well as virtual reality (Lee et al., 2015; Bok et al., 2023) or newer approaches such as LiDAR (Yoon et al., 2021) can be used in the home or community to deliver therapeutic programs and monitor outcomes. However, these methods may have lower spatial and temporal resolution or require additional processing compared to emerging techniques. Newer video-based markerless motion capture approaches can use computer vision and machine learning to estimate human joint positions directly from video, providing powerful tools that non-experts can set up and use. Markerless motion capture using computer vision can provide detailed kinematic measures of gait, posture, and coordination from a simple camera setup. This type of technology has already been used to study movement in people with PD, stroke and cerebral palsy in non-dance contexts (Martinez et al., 2018; Steffensen et al., 2023).

Moreover, while laboratory-based markerless systems - such as Theia3D (Theia Markerless Inc., Kingston, ON, Canada)—often still rely on expensive licensed software, open-source software for motion capture and analysis can remove physical and financial barriers to technology-derived measurement. For example, recent studies in PD have used computer vision analysis (e.g., DeepLabCut, Mediapipe) of pre-recorded videos of hand movements to classify disease state (Heye et al., 2024) and to identify kinematic markers indicating response to levodopa treatment (Lange et al., 2025). These newer motion capture technologies also enable greater flexibility to measure outcomes remotely and at scale.

Existing methods using wearable inertial sensors, smartphones, fitness trackers, and smartwatches can capture movement intensity, gait, and balance during dance activities and daily living (Blackler et al., 2019; Avci, 2024; for review see Tao et al., 2024). In addition, wearable sensors can capture data related to physical activity, falls, sleep patterns, and other physiological indicators of health (e.g., Kristoffersson and Lindén, 2022; Ghazi et al., 2025). However, while most wearable devices currently available are rigid, visible, and can be uncomfortable to wear—limiting their acceptability—the advent of soft smart wearable sensors that conform to the skin and stretch with the body will likely make these technologies more practical and appealing (Kim et al., 2025), thereby increasing the feasibility of longitudinal data collection to investigate longer-term effects of dance.

## Neuroimaging

Neuroimaging methods can be used to detect neuroplastic changes resulting from therapeutic dance in motor-related and other brain regions, complementing behavioral outcome measures. Magnetic resonance imaging (MRI) techniques such as fMRI and diffusion tensor imaging (DTI) have been used to show functional or structural connectivity changes in the brain associated with dance (Teixeira-Machado et al., 2019; Meulenberg et al., 2023; Simon et al., 2024; Tung et al., 2024; Wu et al., 2025). For example, a case study of an individual with PD participating in regular dance classes found functional changes in brain areas involved in movement planning and imagery, rhythm, emotional processing, and multisensory integration (Simon et al., 2024). In addition, dance-based interventions in older adults have been associated with increases in integrity of white-matter tracts and changes in structural connectivity in networks relevant to motor control, balance, and coordination. For example, a study of aerobic dance in older adults with mild cognitive impairment showed enhanced structural connections within the default mode network and between the supplementary motor area and default mode network regions (Wu et al., 2025).

Emerging neuroimaging technologies now have the capacity to record neural activity not only before and after, but also during dance. One such method, functional near-infrared spectroscopy (fNIRS), allows measurement of cortical hemodynamics during movement, enabling more direct investigation of therapeutic mechanisms. For example, an 8-week interactive dance training study in older adults found that changes in prefrontal cortex oxygenation during treadmill walking correlated with improvements in executive functioning (Eggenberger et al., 2016). Mobile electroencephalography (EEG) approaches similarly enable real-time assessment of sensorimotor neural rhythms and event-related desynchronizations during walking (Bonassi et al., 2024), which can also provide insight into mechanisms underlying therapeutic effects of dance, particularly when combined with kinematic measures or correlated with physical function outcomes. Mobile brain/body imaging (MoBI; Barnstaple et al., 2021; King and Parada, 2021) is an example of such an approach, integrating EEG with motion capture during active movement.

## Challenges to integrating new technologies

The integration of new technologies into dance outcome measurement presents several challenges. The rapid pace of digital innovation and AI advances will require ongoing evaluation and updating of assessment tools to optimize validity and reliability. A key issue is the need to establish technology-derived outcomes that are both clinically meaningful and population-specific and can function as biomarkers of therapeutic effects.

Additionally, clinicians and researchers may be hesitant to move away from gold standards, while participants may have reservations about being monitored or having their data collected remotely. Remote assessments also depend on digital literacy, internet connectivity, and access to devices (e.g., Bek et al., 2021; Okafor et al., 2024), and may still require healthcare professionals to assist with setup and training. Finally, cultural differences, such as in attitudes toward technology and privacy, must be considered.

## Future directions

Future research directions will be partly driven by ongoing technological advances. Nonetheless, we can consider some ways in which emerging technologies may contribute to enhancing quantitative outcome measurement in therapeutic dance. With further research and validation, technology-derived measures could identify kinematic or neural “signatures” of improvement in different populations. Remote data collection will enable larger-scale studies to be conducted using home-based training. Open-source software and code sharing could facilitate greater standardization of measurement, leading to more meaningful comparison of outcomes. Moreover, combining technologies such as motion capture and mobile imaging will enable researchers to identify neural correlates of motor function in real-time, to better understand brain mechanisms underlying physical outcomes. Beyond gathering an evidence base to support the development of dance programs and interventions, digital technologies and AI also offer the opportunity to provide personalized feedback and tailored progression informed by dynamic analysis of performance data (e.g., Kang et al., 2023; Liu et al., 2023).

The technologies discussed here in relation to dance can also be applied across many other therapeutic contexts. Facilitating self-monitoring and independent home-based training, where appropriate, can empower patients to take a more active role in managing their rehabilitation (e.g., Kraal et al., 2013; Doyle et al., 2019). Additionally, data can be curated and shared with clinicians and healthcare providers to help guide care.

Further work should cross-validate new measures with gold standards to provide evidence of convergent validity and facilitate meaningful interpretation of novel metrics. For example, computer vision measures of bradykinesia have been validated against clinical ratings of PD symptoms from the MDS-UPDRS (Heye et al., 2024). While digital technology-enabled methods may not replace clinical evaluations and patient-reported outcomes, which will remain important tools in interpreting quantitative metrics relative to contextual factors, they will expand the capacity of clinicians and researchers to precisely, comprehensively, and efficiently evaluate therapeutic dance programs and deliver personalized training.
